# Three-Dimensional Echocardiographic Evaluation of Rheumatic Tricuspid Valve Disease: A Prospective Single-Center Cohort Study

**DOI:** 10.31083/RCM48678

**Published:** 2026-03-06

**Authors:** Mahmoud Abdelnabi, Abdallah Almaghraby, Moustafa Gamal, Mohamed Ayman Abdel-Hay, Hoda Abdelgawad

**Affiliations:** ^1^Department of Cardiovascular Medicine, Mayo Clinic, Phoenix, AZ 85054, USA; ^2^Department of Cardiology, Ibrahim Bin Hamad Obaidallah Hospital, 10190 Ras Al Khaimah, UAE; ^3^Department of Cardiology, Faculty of Medicine, Alexandria University, 5372066 Alexandria, Egypt; ^4^Department of Cardiology, King’s College Hospital NHS Trust, SE5 9RS London, UK

**Keywords:** rheumatic heart disease, tricuspid valve, 2D echocardiography, 3D echocardiography, valvular morphology, commissural fusion, thickness

## Abstract

**Background:**

Rheumatic heart disease (RHD) remains a prevalent cause of valvular heart pathology worldwide, especially in younger populations of low- and middle-income countries. Tricuspid valve (TV) involvement in RHD is usually secondary to left-sided valvular lesions and is often underdiagnosed, since two-dimensional echocardiography (2DE) has limited ability to visualize the complex tricuspid anatomy. Compared with 2DE, three-dimensional echocardiography (3DE) provides an en face visualization of the tricuspid valve, enabling direct planimetric measurements and detailed commissural assessment, and offers advantages for evaluating complex valvular heart disease (VHD). Thus, this study aimed to assess baseline characteristics and valvular morphology in patients with rheumatic tricuspid valve disease using 3DE.

**Methods:**

A prospective cohort single-center study conducted between April 2022 and April 2023 included 34 patients with rheumatic TV involvement. Baseline demographics, morphological features, and hemodynamic parameters were assessed using transthoracic 3DE.

**Results:**

The mean age of included patients was 45.5 ± 9.1 years, and 88.2% were female. Most patients had associated left-sided valvular involvement (mitral or combined mitral and aortic). Commissural fusion (50%), leaflet thickening (82.4%), restricted mobility (85.3%), and coaptation loss (58.8%) were predominantly noted. The mean diastolic TV gradient was 3.9 ± 3.4 mmHg, planimetry area 3.3 ± 1.7 cm^2^, and estimated pulmonary artery systolic pressure 43.3 ± 19.5 mmHg. Mean right ventricular (RV) global longitudinal strain was –23.6 ± 6.1%.

**Conclusion:**

Rheumatic TV involvement is characterized by commissural fusion, leaflet thickening, and coaptation loss, highlighting the diagnostic value of 3DE in identifying morphological patterns that may guide intervention planning.

## 1. Introduction

Rheumatic heart disease (RHD) remains a significant cause of valvular heart disease globally, particularly affecting children and young adults in resource-limited regions [1]. While RHD predominantly affects mainly the mitral valve (and to a lesser extent the aortic valve), the tricuspid valve (TV) involvement in RHD is less common than left-sided valve involvement, but it is not rare, with a reported prevalence of 7–9% by echocardiography and up to 22–44% in autopsy studies. It almost always occurs in association with mitral valve disease, particularly severe mitral stenosis, and is rarely isolated [2]. Nonetheless, accurate identification of rheumatic TV pathologies during comprehensive echocardiographic evaluation to inform optimal therapeutic decision-making. Two-dimensional echocardiography (2DE) is the standard initial diagnostic test for patients with known or suspected valvular heart disease (VHD) [3]. However, 2DE is limited by its inability to visualize all three TV leaflets simultaneously and by view-dependent variability, which can lead to underestimation of annular size and incomplete assessment of leaflet pathology and regurgitant jets [4, 5]. Three-dimensional echocardiography (3DE) provides an incremental diagnostic value, especially for complex anatomy, procedural planning, and accurate quantification of tricuspid stenosis and regurgitation (TS, TR) severity [1, 6]. En face view enables visualization of all three leaflets and the entire annulus in a single view, allowing for more precise assessment of leaflet pathology, annular dimensions, TR possible mechanism, and the effective regurgitant orifice area and vena contracta area. Additionally, 3DE multiplanar reconstruction (MPR) enables planimetry-derived valve area [1, 6]. This cohort study aimed to describe morphological and hemodynamic abnormalities of TV in patients with rheumatic TV involvement using 3DE.

## 2. Materials and Methods 

### 2.1 Study Design and Population

This was a prospective single-center observational study conducted at the department of Cardiology, Faculty of Medicine, Alexandria University, a tertiary academic medical center in Alexandria, Egypt from April 2022 to April 2023. All participants provided informed consent, and the study protocol was approved by the institutional ethics committee (Approval Number: 0107501/2022), and all procedures were performed in accordance with the Declaration of Helsinki. Thirty-four consecutive patients with echocardiographically confirmed RHD and TV involvement were enrolled. Inclusion criteria included a history of rheumatic fever or definite RHD affecting at least one left-sided valve, plus echocardiographic evidence of TV morphological abnormalities consistent with RHD (such as leaflet thickening, retraction, or commissural fusion). Patients with primary non-rheumatic TV disease (e.g., carcinoid syndrome, infective endocarditis, or congenital Ebstein anomaly) were excluded. All participants provided informed consent, and the local institutional review board approved the study protocol.

### 2.2 Echocardiographic Assessment 

All patients underwent comprehensive 2D and 3D transthoracic echocardiography (Philips EPIQ CVx ultrasound system with X5-1 matrix-array transducer). Standard parasternal, apical, and RV inflow views were acquired with 3D full-volume data sets optimized for temporal and spatial resolution. Two experienced echocardiographers performed all measurements, and any discrepancies were resolved by consensus.

Parameters included:

- Morphological assessment including multivalvular involvement with specific focus on TV, as follows: leaflet thickening, commissural fusion, calcification, loss of coaptation, and chordal thickening.
- Hemodynamic assessment including trans-tricuspid flow velocities and mean diastolic gradients, planimetry-derived valve area through MPR of 3DE “en face” view of the tricuspid orifice in maximal diastolic opening, severity of TR, and pulmonary artery systolic pressure (PASP).
- Right ventricular function assessment: right atrial and ventricular dimensions and RV global longitudinal strain (RVGLS) by 2D speckle-tracking from RV-focused apical view.

### 2.3 Statistical Analysis

Data are presented as mean ± standard deviation (SD) for continuous variables and categorical variables as counts and frequencies for categorical variables. Analyses were performed using SPSS v. 25.0 (IBM-SPSS Statistics, Chicago, IL, USA). Given the study’s descriptive design, no formal hypothesis testing was conducted due to the exploratory nature of this small cohort.

## 3. Results

### 3.1 Baseline Characteristics

A total of 34 patients with rheumatic tricuspid valve involvement were included. The cohort was predominantly middle-aged women (mean age 45.5 ± 9.1 years; 88.2% female). Dyspnea (New York Heart Association (NYHA) II–III) was the predominant symptom (76.5%).

### 3.2 Echocardiographic and Hemodynamic, and Functional Parameters

**Fig. 1.**
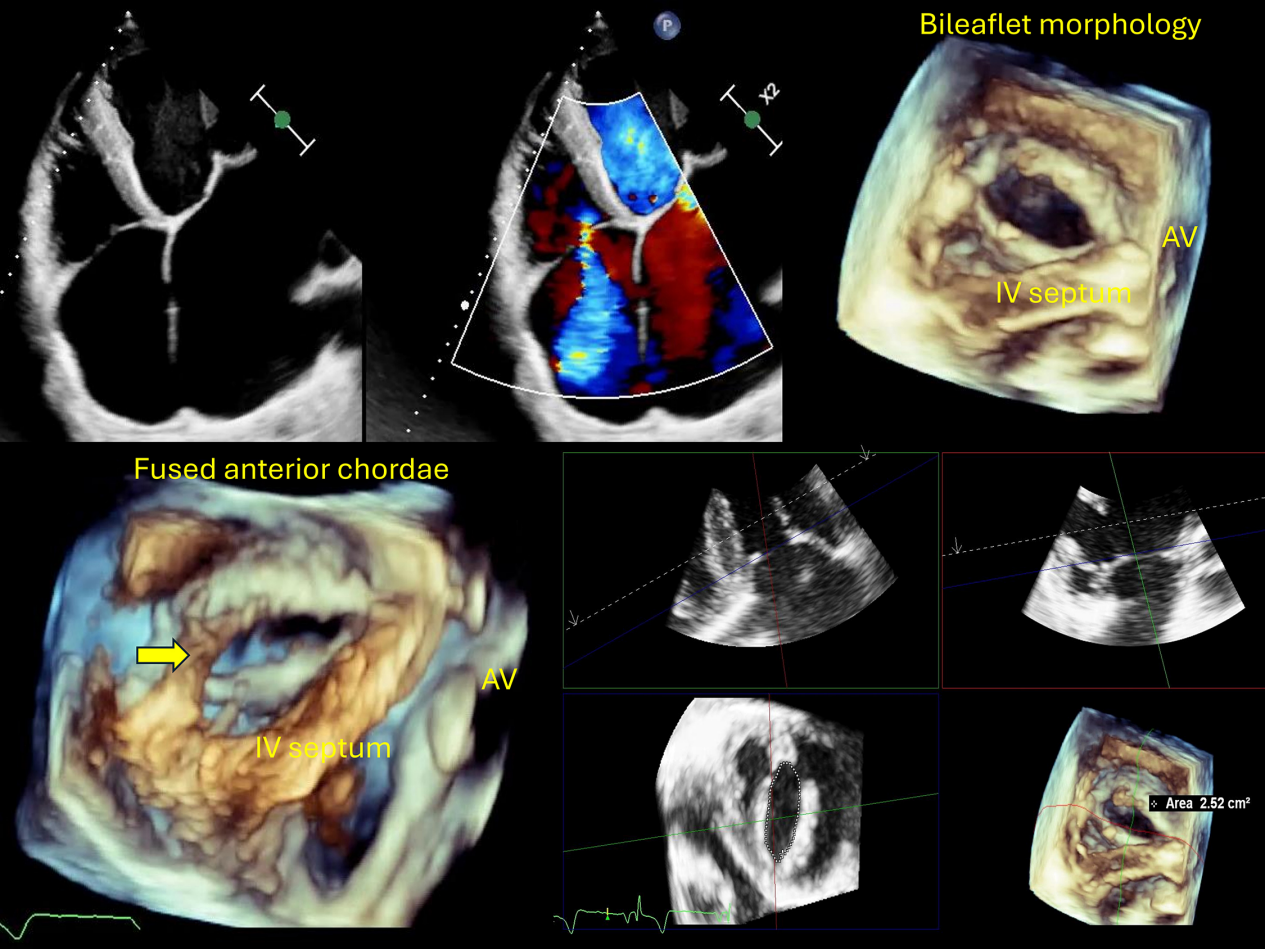
**2D transthoracic echocardiography (TTE) showed mildly thickened leaflets and moderate central tricuspid regurgitation while 3D TTE demonstrated morphologically bi-leaflet valve (type II, septal, and non-septal configuration) with a fused anteroposterior commissure [7]**. Multiplanar reconstruction, aligning the slicing planes at the valve ostium in maximum diastole, revealed an area of 2.52 cm^2^ by direct planimetry. Additionally, thickened and fused chordae were clearly visualized suggestive of rheumatic tricuspid regurgitation without significant stenosis. The yellow arrow indicates the fused anterior chordae. 2D, two-dimensional; 3D, three-dimensional.

**Fig. 2.**
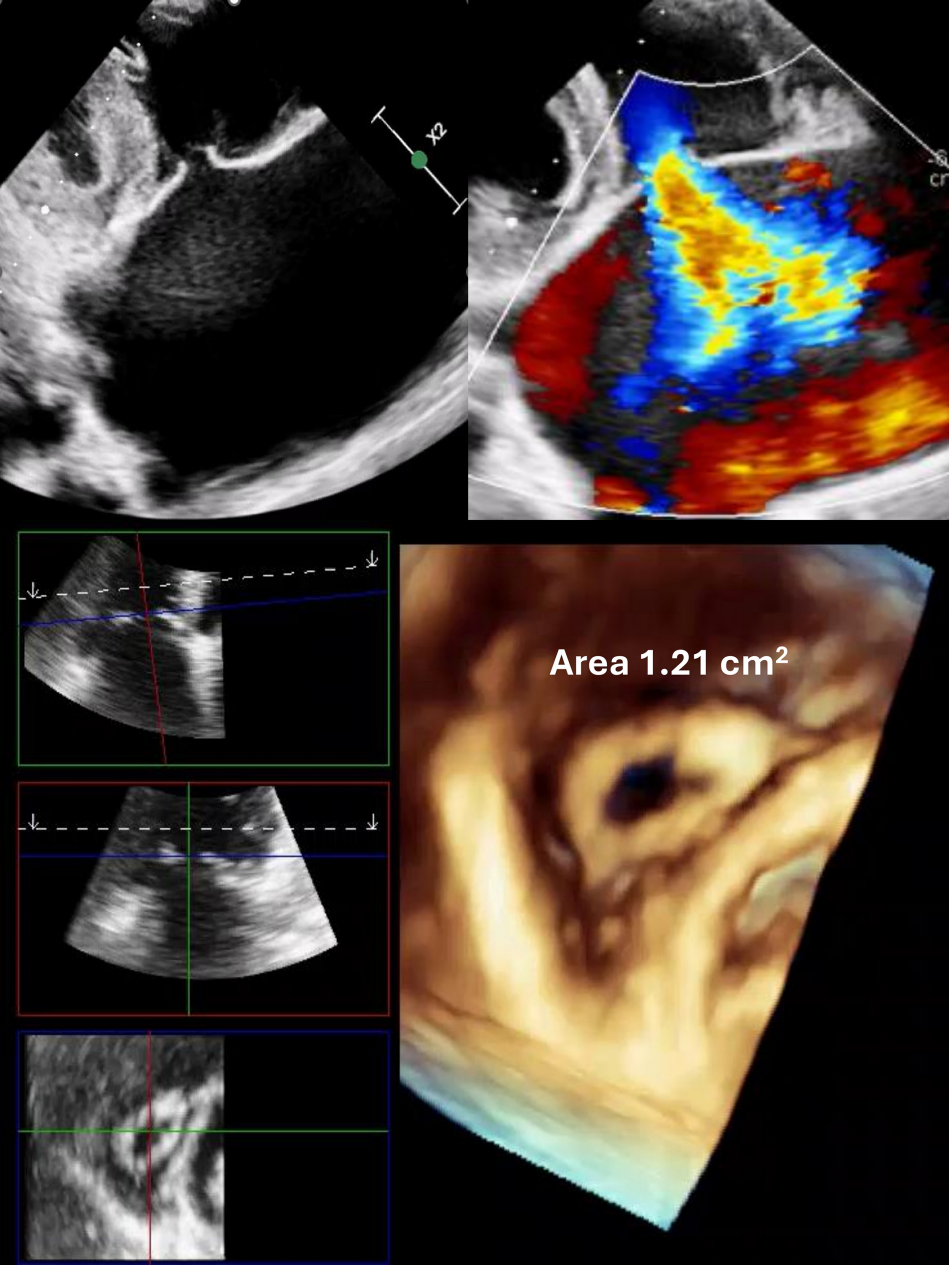
**2D transthoracic echocardiography (TTE) showed thickened leaflets and loss of coaptation with severe torrential tricuspid regurgitation while 3D TTE showing tricommissural fusion**. Multiplanar reconstruction, aligning the slicing planes at the valve ostium in maximum diastole, revealed an area of 1.21 cm^2^ by direct planimetry. Additionally, thickened and fused chordae were clearly visualized suggestive of severe rheumatic tricuspid regurgitation and stenosis.

Associated valvular involvement was frequent (either mitral or combined mitral and aortic involvement). Overall, leaflet thickening (grade 1–3) was present in 28 of 34 patients (82.4%), restricted leaflet mobility (grade 1–3) was observed in 29 of 34 patients (85.3%), commissural fusion was present in 17 of 34 patients (50.0%), most commonly involving the anteroposterior (AP) commissure in 10 patients (29.4%), loss of coaptation was present in 20 patients (58.8%). TV diastolic gradient averaged 3.9 ± 3.4 mmHg, and planimetry area was 3.3 ± 1.7 cm^2^. Estimated pulmonary artery systolic pressure was 43.3 ± 19.5 mmHg, and right ventricular global longitudinal strain was –23.6 ± 6.1%. (Table 1 summarizes detailed morphological and hemodynamic findings. Figs. 1,2 (Ref. [7]) illustrate examples of included cases, Fig. 1 shows moderate rheumatic tricuspid regurgitation (valve area 2.52 cm^2^), and Fig. 2 shows severe rheumatic tricuspid disease with torrential regurgitation and stenosis (valve area 1.21 cm^2^).

**Table 1.** **Baseline characteristics, valvular morphologic and hemodynamic findings**.

Age (years)		45.5 ± 9.1
Gender (Female)		30 (88.2%)
Associated Rheumatic Valves		
	Mitral	17 (50%)
	Aortic	0 (0%)
	Both Mitral and Aortic	17 (50%)
Valve Morphology Type		
	Type 1	13 (38.2%)
	Type 2	15 (44.1%)
	Type 3	6 (17.7%)
Thickness		
	0	6 (17.7%)
	1	14 (41.2%)
	2	10 (29.4%)
	3	4 (11.8%)
Mobility		
	0	5 (14.7%)
	1	15 (44.1%)
	2	8 (23.5%)
	3	6 (17.7%)
Calcification		0 (0%)
Loss of Coaptation		20 (58.8%)
Commissural Fusion		
	None	17 (50.0%)
	AP	10 (29.4%)
	AS	2 (5.9%)
	PS	2 (5.9%)
	AP + AS	1 (2.9%)
	AP + PS	0 (0%)
	AS + PS	1 (2.9%)
	AP + AS + PS	1 (2.9%)
Mean Diastolic Gradient (mmHg)		3.9 ± 3.4
Area by Planimetry (cm²)		3.3 ± 1.7
TR Severity		
	None	2 (5.9%)
	Trace	4 (11.8%)
	Mild	9 (26.5%)
	Moderate	8 (23.5%)
	Severe	11 (32.4%)
Estimated PASP (mmHg)		43.3 ± 19.5
RVGLS (%)		–23.6 ± 6.1

Abbreviations: AP, anteroposterior; AS, anteroseptal; PS, posteroseptal; PASP, pulmonary artery systolic pressure; RVGLS, right ventricular global longitudinal strain; TR, tricuspid regurgitation. Data presented as mean ± standard deviation or number (count and percentage).

## 4. Discussion

In this cohort prospective study of 34 patients with RHD and TV involvement. A detailed characterization of TV morphology was provided using 3DE. Our patient population was predominantly composed of middle-aged women with multivalvular RHD (most commonly mitral stenosis or combined stenosis and regurgitation), and many had concomitant aortic valve lesions, which concurs with previous data that isolated rheumatic TV involvement is rare [2]. Rheumatic TV disease in our cohort was characterized by marked commissural fusion—particularly at the AP commissure—along with variable degrees of leaflet thickening, restricted mobility, and frequent chordal fusion, which would have been difficult to determine by 2DE due to the lack of a reliable short-axis view, while the en face 3D view can provide a “surgical” perspective of the valve [8], allowing direct visualization of fused commissures, most commonly the AP commissure, resulting in a bileaflet valvular morphology in many patients, with more advanced cases showing even complete three-commissural fusion. Many patients demonstrated significant TR due to leaflet coaptation, restriction, and chordal fusion despite a stenotic orifice, indicating a mixed lesion (TS and TR) and suggesting that TR was driven by organic rheumatic TV leaflet pathology rather than functional annular dilation. MPR 3DE reconstruction enables direct planimetry and detects TS cases that might have been underestimated by 2DE by low Doppler gradients, likely due to reduced forward flow in the presence of significant TR. This confirms that 2D-Doppler alone can underestimate TS severity, while 3D planimetry provides a more accurate, flow-independent measurement [6]. Overall, our findings confirm that 3DE has incremental diagnostic value over 2D imaging in valvular RHD, improving recognition of commissural fusion and TS severity and clarifying TR mechanisms—essential for guiding surgical planning or future transcatheter interventions. Previous data on rheumatic TV diseases are scarce, with previous evidence being derived from case reports, series, and small cohort studies. Pothineni *et al*. [9] showed that in a study of 29 patients with diverse TV pathologies, live/real-time transthoracic 3DE allowed “en face” visualization of all three leaflets, accurate assessment of orifice area, identification of leaflet defects, and quantification of TR severity, offering substantial incremental diagnostic value over 2DE for evaluating a broad range of TV abnormalities. Additionally, previous case reports highlighted the added value of 3DE in evaluating TV stenosis. Anwar *et al*. [10] demonstrated that real-time 3DE provides superior visualization of individual leaflets and commissures, allowing accurate planimetry of the TV area and better discrimination between rheumatic stenosis and normal or non-stenotic valves. Abdelnabi *et al*. [7] supported these findings, showing that 3DE reliably identifies commissural fusion, chordal thickening, and subvalvular involvement while distinguishing rheumatic pathology from mimickers such as carcinoid TV disease. Future studies of rheumatic TV disease should integrate comprehensive 3D assessment of the RV function, including RV–pulmonary artery coupling metrics such as the stroke volume to RV end-systolic volume ratio, which has demonstrated strong correlation with invasive pressure–volume–derived indices and may further refine risk stratification and management, particularly in patients with severe tricuspid regurgitation [11].

## 5. Limitations

The cohort study has several limitations. First, this was a single-center study with a small sample size and no longitudinal follow-up; however, to the best of our knowledge, this was one of the largest cohorts to focus on 3DE assessment of rheumatic TV. Second, there is no comparative gold standard for TV evaluation; however, TV 3D planimetry is arguably the best available noninvasive modality. Third, morphological features assessment, such as grading leaflet thickening or extent of commissural fusion, was somewhat subjective, though performed by experienced readers; developing standardized 3D scoring for TV morphological abnormalities should be an area for future research. Fourth, clinical follow-up and outcomes were not reported in this short communication. Finally, while 3DE offers important incremental anatomic and diagnostic information in TV disease, the present study didn’t evaluate cost-effectiveness, which remains an important consideration for broader clinical implementation. Large multi-center studies are warranted to validate 3DE-derived predictors (e.g., degree of commissural fusion or measured valve area) of long-term clinical outcomes or surgical findings. Large multi-center studies are warranted to validate 3DE-derived predictors (e.g., degree of commissural fusion or measured valve area) of long-term clinical outcomes or surgical findings and cost effectiveness in comparison to 2DE.

## 6. Conclusions

Rheumatic tricuspid valve disease is characterized by commissural fusion, leaflet thickening, and loss of coaptation, predominantly in middle-aged women with multivalvular RHD. 3DE provides comprehensive morphological and functional insights that enhance diagnostic accuracy and procedural planning. Future research should explore 3DE’s role in risk stratification and therapeutic planning.

## Data Availability

The datasets used and analyzed during the current study are available from the corresponding author on reasonable request.

## Abbreviations

2DE, two-dimensional echocardiography; 3DE, three-dimensional echocardiography; AP, anteroposterior; AS, anteroseptal; PS, posteroseptal; CM^2^, square centimeters; MPR, multiplanar reconstruction; NYHA, New York Heart Association; PASP, pulmonary artery systolic pressure; RA, right atrium; RV, right ventricle; RV-GLS/RVGLS, right ventricular global longitudinal strain; RHD, rheumatic heart disease; SPSS, statistical package for the social sciences; SD, standard deviation; TR, tricuspid regurgitation; TS, tricuspid stenosis; TV, tricuspid valve; VHD, valvular heart disease.
